# Reply: On the Reporting of Odds Ratios and Risk Ratios, *Nutrients* 2018, *10*, 10

**DOI:** 10.3390/nu10111581

**Published:** 2018-10-26

**Authors:** Elena Ricci, Stefania Noli, Sonia Cipriani, Irene La Vecchia, Francesca Chiaffarino, Stefania Ferrari, Paola Agnese Mauri, Marco Reschini, Luigi Fedele, Fabio Parazzini

**Affiliations:** 1 Dipartimento Donna-Bambino-Neonato, Fondazione IRCCS Ca’ Granda Ospedale Maggiore Policlinico, 20122 Milan, Italy; son.cipriani@gmail.com (S.C.); francesca.chiaffarino@gmail.com (F.C.); stefania.ferrari@policlinico.mi.it (S.F.); paola.mauri@unimi.it (P.A.M.); luigi.fedele@unimi.it (L.F.); fabio.parazzini@unimi.it (F.P.); 2 Department of Clinical Sciences and Community Health, Università di Milano, Fondazione IRCCS Ca’ Granda Ospedale Maggiore Policlinico, 20122 Milan, Italy; stefi.noli@gmail.com (S.N.); irene.lavecchia@unimi.it (I.L.V.); 3 Infertility Unit, Fondazione IRCCS Ca’ Granda Ospedale Maggiore Policlinico, 20122 Milan, Italy; m.reschini@gmail.com

## Dear Editor,

In response to the letter of Pace and Multani, in general, we cannot disagree with their considerations about the use of odds ratios, risk ratios, and rate ratios.

In the first version of the paper, our results were reported as odds ratios. Dr. Pace, one of the two reviewers, requested a new analysis more adequate to the prospective design of the study. Although all estimates were not far from unit and odds ratios were good estimates of both risk ratios and rate ratios, we agreed to that request and reported our results as rate ratios. 

It was not the first time that, in the framework of assisted reproduction techniques, rate ratios have been calculated instead of risk ratios. Such a use is due to the fact that, implicitly, a cycle of ART is considered a unit of time, thus leading to the consideration of the proportion of failure or success as a rate [1,2]. In such a situation, rate ratios and risk ratios are equal. 

The conclusions of our study did not change when we changed the analysis: Odds ratios and rate ratios for the three outcomes are reported in the Table 1. What we forgot to modify was the statistical methods, where “logistic regression” was left instead of “Cox regression”. For this reason, we apologize both to the Journal and to the readers.

We should have been more explicit about these aspects of the statistical methodology, but, as the revised version was approved and published, we thought that it was clear enough. Presumably, Dr. Pace did not even see the second version, or he would have asked a further revision. 

## Figures and Tables

**Table 1:** Odds ratios and rate ratios for the three outcomes.

	Implantation		Clinical Pregnancy		Live Birth	
**Women**	**AOR (95% CI)**	**ARR (95% CI)**	**AOR (95% CI)**	**ARR (95% CI)**	**AOR (95% CI)**	**ARR (95% CI)**
**Caffeine intake**						
1st tertile	1	1	1	1	1	1
2nd tertile	1.42 (0.62–3.23)	1.34 (0.64–2.79)	1.23 (0.66–2.29)	1.07 (0.76–1.50)	1.50 (0.74–3.02)	1.09 (0.79–1.50)
3rd tertile	0.86 (0.34–2.19)	0.90 (0.38–2.10)	0.97 (0.52–1.84)	1.00 (0.70–1.43)	0.94 (0.47–1.88)	0.99 (0.71–1.40)
>90° percentile ^§^	0.49 (0.12–2.05)	0.58 (0.16–2.04)	0.80 (0.25–2.59)	0.96 (0.51–1.80)	0.90 (0.27–3.06)	0.99 (0.54–1.84)
**Men**						
**Caffeine intake**						
1st tertile	1	1	1	1	1	1
2nd tertile	1.86 (0.82–4.23)	1.64 (0.78–3.44)	1.02 (0.56–1.87)	1.01 (0.72–1.42)	1.11 (0.56–2.19)	1.02 (0.75–1.41)
3rd tertile	0.74 (0.29–1.92)	0.78 (0.32–1.87)	1.28 (0.68–2.43)	1.07 (0.76–1.52)	1.02 (0.51–2.05)	1.00 (0.72–1.40)
>90° percentile ^§^	0.68 (0.14–3.39)	0.73 (0.17–3.16)	1.48 (0.47–4.61)	1.12 (0.59–2.14)	1.00 (0.29–3.46)	0.98 (0.54–1.83)
**Combined intake (W-M)**						
Low-low	1	1	1	1	1	1
Low-high	0.75 (0.29–1.91)	0.79 (0.34–1.85)	1.12 (0.54–2.32)	1.03 (0.71–1.50)	1.51 (0.67–3.40)	1.09 (0.76–1.55)
High-low	1.28 (0.51–3.22)	1.22 (0.53–2.78)	0.85 (0.41–1.74)	0.95 (0.64–1.42)	1.24 (0.55–2.80)	1.06 (0.73–1.54)
High-high	0.54 (0.20–1.44)	0.60 (0.24–1.48)	0.70 (0.36–1.36)	0.89 (0.61–1.30)	0.74 (0.36–1.52)	0.93 (0.65–1.33)

AOR: adjusted odds ratio; ARR: adjusted rate ratio; CI: confidence interval; W-M: women-men; ^§^ as compared to 10th percentile.
